# Supplementary material for: Content-rich biological network constructed by mining PubMed abstracts
Source: BMC Bioinformatics. 2004 Oct 8;5:147. doi: 10.1186/1471-2105-5-147 (PMC528731; doi:10.1186/1471-2105-5-147)
Supplement: Additional File 2 — The original results of the above study (non-essential files are deleted to keep the file size under the limit set by BMC bioinformatics). [file 1471-2105-5-147-S2.bz2 › chilibotAdditionalFile2/dip10/614ID10619841E10997/html/GTF2B_TBP.html]

 


 **GTF2B** and **TBP** 
  
Found 354 abstracts in PubMed, retrieved 10.  
 

 What does Google say? 
 PDF only 
| .edu only 

---

**Interactive relationship** (e.g. stimulation, inhibition, etc)

**Neutral relationship**- The interacting region identified in IE62 overlaps with or borders on sites involved in interactions with the VZV IE4 protein and the cellular factors  **TBP**  and TFIIB  [ **GTF2B** ] .  Ref: 12855699 J Biol Chem< MedlineTA>J Biol Chem,
- This is accomplished by the ability of the TFIIIB subunit, TFIIB  [ **GTF2B** ]  related factor Brf1, to make stable protein protein interactions with TATA binding protein  [ **TBP** ]   **TBP**  and place it on the promoter less upstream DNA.  Ref: 12682361 Nucleic Acids Res, 2003
- The fission yeast TFIIB  [ **GTF2B** ]  related factor limits RNA polymerase III to a TATA dependent pathway of  **TBP**  recruitment.  Ref: 12682361 Nucleic Acids Res, 2003
- Taken together, our results indicate that the spatial positioning of the DNA bound activation domain is important for efficient activation, possibly by maximizing its interactions with the transcriptional machinery including the  **TBP**  TFIIA TFIIB  [ **GTF2B** ]  promoter quaternary complex.  Ref: 12538582 J Biol Chem, 2003
- Interactions of a DNA bound transcriptional activator with the  **TBP**  TFIIA TFIIB  [ **GTF2B** ]  promoter quaternary complex.  Ref: 12538582 J Biol Chem, 2003
- This result implies DNA bending and looping of promoter DNA as a result of the physical interaction between GAL4 VP16 and an interface of the  **TBP**  TFIIA TFIIB  [ **GTF2B** ]  complex.  Ref: 12538582 J Biol Chem, 2003
- This protein protein interaction on promoter DNA minimally requires the presence of one GAL4 binding site and the formation of a quaternary complex containing  **TBP** , TFIIB  [ **GTF2B** ] , and TFIIA on the TATA element.  Ref: 12538582 J Biol Chem, 2003
- Interaction of the equine herpesvirus 1 EICP0 protein with the immediate early IE protein, TFIIB  [ **GTF2B** ] , and  **TBP**  may mediate the antagonism between the IE and EICP0 proteins.  Ref: 12552007 J Virol, 2003
- Notably, the topology of the  **TBP**  TFIIA TFIIB  [ **GTF2B** ]  promoter complex is not altered significantly by the interaction with DNA bound activators.  Ref: 12538582 J Biol Chem, 2003
- Taken together, these results suggested that interaction of the EICP0 protein with the IE protein, TFIIB  [ **GTF2B** ] , and  **TBP**  may mediate the antagonism between the IE and EICP0 proteins.  Ref: 12552007 J Virol, 2003
- In vitro interaction assays revealed that the EICP0 protein interacted directly with the basal transcription factors TFIIB  [ **GTF2B** ]  and  **TBP** .  Ref: 12552007 J Virol, 2003
- Our simulated TATA  **TBP**  complexes develop sequence dependent structure and motion trends that may lead to favorable orientations for high activity variants with respect to binding TFIIA, TFIIB  [ **GTF2B** ] , and other transcription factors, while conversely, accelerate dissociation of low activity TATA  **TBP**  complexes.  Ref: 12767124 Biopolymers, 2003
- We also found through chromatin immunoprecipitation experiments that the interaction between NZFP and  **TBP**  inhibits binding of TFIIA and TFIIB  [ **GTF2B** ] .  Ref: 12788093 Biochem Biophys Res Commun, 2003
- A novel  **TBP**  interacting zinc finger protein represses transcription by inhibiting the recruitment of TFIIA and TFIIB  [ **GTF2B** ] .  Ref: 12788093 Biochem Biophys Res Commun, 2003
- These data strongly suggest that the repression by NZFP occurs through its binding to both DNA and  **TBP**  and the resulting NZFP  **TBP**  promoter complex inhibits preinitiation complex assembly by preventing binding of TFIIA and TFIIB  [ **GTF2B** ] .  Ref: 12788093 Biochem Biophys Res Commun, 2003

**Non-interactive relationship** (e.g. studied together, co-existance, homology, etc.)

- Native human TATA binding protein  [ **TBP** ]  simultaneously binds and bends promoter DNA without a slow isomerization step or TFIIB  [ **GTF2B** ]  requirement.  Ref: 12791683 J Biol Chem< MedlineTA>J Biol Chem, 2003
- The inhibitory effect of E1A 13S on the assembly of the PIC is overcome when excess  **TBP**  is added back in the reaction, while addition of either excess TFIIA or TFIIB  [ **GTF2B** ]  were ineffective.  Ref: 12818428 Biochim Biophys Acta, 2003
- Transcription factor IIIB TFIIIB, consisting of the TATA binding protein  [ **TBP** ]   **TBP** , TFIIB  [ **GTF2B** ]  related factor Brf1 and Bdp1, is a central component in basal and regulated transcription by RNA polymerase III.  Ref: 12660736 Nature, 2003
- In addition, EMSA studies show that adenovirus E1A 13S oncoprotein is unable to disrupt either the preestablished TFIIA  **TBP**  TATA or TFIIA TFIIB  [ **GTF2B** ]   **TBP**  TATA complexes, but does inhibit complex formation when all transcription factors were simultaneously added.  Ref: 12818428 Biochim Biophys Acta, 2003
- Site specific protein DNA photo cross linking was used to show that, when bound to its cognate site at various distances upstream of the TATA element, the chimeric transcriptional activator GAL4 VP16 can physically interact with a TATA box binding protein  [ **TBP** ]   **TBP**  transcription factor IIA TFIIA TFIIB  [ **GTF2B** ]  complex assembled on the TATA element.  Ref: 12538582 J Biol Chem, 2003
- The amino terminal half of Brf1 shares a high degree of sequence similarity with the polymerase II general transcription factor TFIIB  [ **GTF2B** ] , but it is the carboxy terminal half of Brf1 that contributes most of its binding affinity with  **TBP** .  Ref: 12660736 Nature, 2003
- Our unpublished data showed that the IE protein interacts with the same domain aa 125 to 174 of TFIIB  [ **GTF2B** ]  and with  **TBP** .  Ref: 12552007 J Virol, 2003
- Chromatin immunoprecipitation analyses demonstrated that T cell activation triggers in vivo binding of endogenous c Rel, c Jun, C EBP beta, and HMG IC to the bfl 1 regulatory region, coincident with selective recruitment of coactivators TAFII250 and p300, SWI SNF chromatin remodeling factor component BRG 1, and basal transcription factors TATA binding protein  [ **TBP** ]   **TBP**  and TFIIB  [ **GTF2B** ] , as well as hyperacetylation of histones H3 and H4.  Ref: 12665576 Mol Cell Biol, 2003
